# On the use of deep learning for computer-generated holography

**DOI:** 10.1016/j.isci.2025.112507

**Published:** 2025-04-23

**Authors:** Xuan Yu, Haomiao Zhang, Zhe Zhao, Xuhao Fan, Shaodong Hu, Zongjing Li, Wenbin Chen, Daqian Li, Shaoxi Shi, Wei Xiong, Hui Gao

**Affiliations:** 1 Wuhan National Laboratory for Optoelectronics and School of Optical and Electronic Information, Huazhong University of Science and Technology, Wuhan, Hubei 430074, China; 2 Zhejiang University, Hangzhou, Zhejiang 310027, China; 3 School of Engineering, Westlake University, Hangzhou, Zhejiang 310030, China; 4 Optics Valley Laboratory, Wuhan, Hubei 430074, China

**Keywords:** Computer science, Physics

## Abstract

The research disciplines of computer-generated holography (CGH) and machine learning have evolved in parallel for decades and experienced booming growth due to breakthroughs in mathematical optimization and computing hardware. Over the past few years, deep learning has been applied to CGH and achieved remarkable success, accustoming a great step toward high-quality and real-time holographic display. This review introduces the fundamental concepts of CGH and deep learning, examines the development of deep-learning–based computer-generated holography (DLCGH), and explores cutting-edge research frontiers including data-driven models, physics-driven models, and jointly optimized models. Finally, we summarize with an outlook on the challenges and prospects of DLCGH.

## Graphical abstract

**Figure:**
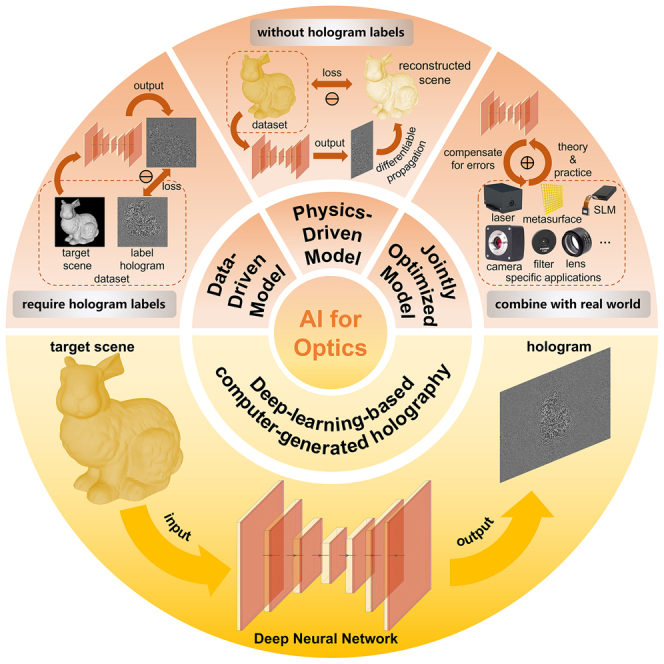

## Introduction

As one of the most promising candidates for next-generation stereo display approach, computer-generated holography (CGH) can provide all visual cues through faithfully recording optical field information with numerical calculation,^1^^,^^2^ which plays a pivotal role in diverse display domains such as augmented reality (AR),^3^ virtual reality (VR),^4^^,^^5^ 3D projection,^6^ and metaverse.^7^ However, conventional numerical calculation methods entail significant computational burdens while struggling to balance computing speed and image quality, thereby impeding the advancement of CGH. In recent years, advances in high performance computing hardware such as graphics processing units (GPUs) and tensor processing units (TPUs) have revolutionized AI, especially deep learning, which is profoundly altering the whole world. The remarkable progress in deep learning has not only permeated diverse practical domains including large language models,^8^ computer vision^9^^,^^10^ and meteorological forecasting,^11^ but also sparked a paradigm shift across scientific disciplines through the “AI for Science” movement.^12^ Focusing on AI for optics, deep learning has boosted computational imaging^13^^,^^14^ and optical computing,^15^^,^^16^ while simultaneously opening new methodological possibilities for CGH^17^ and leading to astonishing achievements.

**Figure 1:**
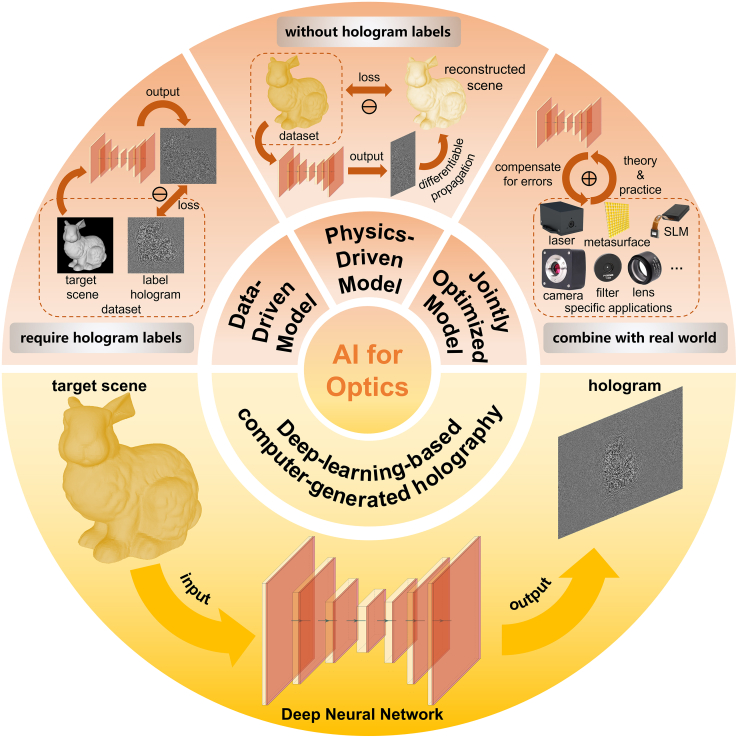
Overview of deep-learning–based computer-generated holography Data-driven models require labels to establish precise mapping relationships between the input scenes and output holograms. In contrast, physics-driven models generate new holograms without label holograms as training data because the generated holograms are propagated to the viewing position via differentiable physical algorithms and then the loss function is directly computed between simulated reconstructions and target scenes. Jointly optimized models focus on application-specific implementations by synergistically combining optical components with neural networks to mitigate discrepancies between theoretical predictions and experimental constraints.

In this review, we systematically outline recent advancements and persistent challenges in deep-learning–based computer-generated holography (DLCGH). We provide a concise overview of fundamental principles encompassing both CGH and deep learning methodologies, covering classical CGH algorithms, representative neural network architectures, and contemporary training paradigms. Through comprehensive analysis, we examine current advances in DLCGH across three primary frameworks: data-driven models, physics-driven models, and jointly optimized models, as illustrated in Figure 1. Finally, we critically discuss emerging challenges and propose future research directions to advance DLCGH development.

## Basic principles of CGH and deep learning

### A brief outline of CGH

Optical holography was initially invented by Denis Gabor to enhance the resolution of electron microscope,^18^ a breakthrough that earned him the 1971 Nobel Prize in Physics. This technology predominantly contains two steps: (1) recording the wavefront of the object into a media (i.e., a hologram) using light interference, and (2) illuminating the hologram to reconstruct two-dimensional (2D) or three-dimensional (3D) images of the object. Stemming from the original principles of holography, CGH uses computational algorithm in place of optical recording to generate the hologram, eliminating reliance on real-scene 3D objects and sophisticated optical setups. The mathematical model of CGH can be represented as:

(Equation 1) $$I_{\text{tar}}=P\left(\Phi \right)\text{,}$$

(Equation 2) $$\Phi =P^{-1}\left(I_{\text{tar}}\right)\text{,}$$

where $I_{\text{tar}}$ is known target image, P represents the optical propagation, and we aim to find the desired hologram Φ inversely, which may yield multiple solutions.

To solve the abovementioned ill-posed inverse problem, traditional CGH algorithms have proliferated and are broadly categorized into two main types: iterative and non-iterative approaches. Iterative algorithms involve Gerchberg-Saxton (GS),^19^ Wirtinger holography,^20^ gradient descent^21^^,^^22^ and their variants. With regard to non-iterative algorithms, double phase-amplitude coding (DPAC)^23^ and its variants enable fast hologram generation in fewer steps. For 3D CGH, there are point-based,^24^^,^^25^^,^^26^ polygon-based,^27^ and layer-based^28^ method, etc., which are based on various elements decomposition but require a significant amount of computing resources. Once calculated, the hologram should be loaded onto optical modulation devices such as spatial light modulator (SLM),^29^ digital micromirror device (DMD),^30^ and metasurface^31^^,^^32^^,^^33^ for image reconstruction. Depending on different modulation types, CGH can compute complex holograms, amplitude-only holograms, or phase-only holograms (POHs) for display. While complex holograms theoretically achieve optimal reconstruction fidelity, they require sophisticated algorithm design and experimental setups.^34^ Amplitude-only holograms, which only modulate amplitude information, exhibit substantial energy loss leading to reduced diffraction efficiency and brightness in reconstructed images.^35^ In contrast, POH optimization has been extensively investigated on account of higher diffraction efficiency and excellent compatibility with commercial SLMs based on the assumption that reconstruction can be achieved with only the phase information of scattered wavefront.^36^^,^^37^

While traditional algorithms have laid a critical foundation for CGH, they often face limitations in balancing reconstruction fidelity and computational efficiency. Iterative approaches, notably the GS algorithm, demonstrate progressive enhancement in reconstructed image quality with increasing iterations. However, the intrinsic speckle noise originating from initial random phase persists regardless of iteration count,^38^ while slow convergence rates necessitate substantial computational expenditure. Conversely, non-iterative methods such as DPAC excel in computational efficiency but are prone to generating pronounced artifacts near high-frequency components and occluded boundaries in complex scenes.^39^ Besides, both iterative and non-iterative methods require complete recomputation for new input scenes, incurring substantial computational overhead. These challenges are exacerbated in dynamic or real-time applications, such as holographic displays for AR and VR.

To overcome these bottlenecks, deep learning has emerged as a paradigm-shifting alternative. DLCGH have demonstrated high-speed and high-quality performance, holding great promise for next-generation holography.

### Fundamentals of deep learning for CGH

Machine learning, a cornerstone of AI, has achieved significant breakthroughs in autonomously enhancing algorithm performance by leveraging real-world data and prior experiential knowledge. This computational paradigm emulates human learning processes through artificial neural networks,^40^^,^^41^ with its profound impact acknowledged by the 2024 Nobel Prize in Physics. As the most advanced evolution of machine learning, deep learning is designed to learn the inherent rules and hierarchical representations from sample data, exhibiting unprecedented analytical capabilities that enable machines to perform more complicated tasks like humans.^42^^,^^43^ The technical foundation resides in deep neural networks (DNNs), characterized by multiple hidden layers between the input and output nodes, which process information through learnable weight parameters and nonlinear activation functions. Essentially, DNNs combine multiple adaptive function layers into sophisticated computational systems capable of handling diverse tasks. The fundamental objective involves finding network parameters that minimize the discrepancy between predicted outputs and ground truth values, constituting a multidimensional optimization problem. Given a dataset (X,Y), DNN maps input X to predicted output $\hat{Y}$, then the network parameters are optimized to fit the label value Y,which can be described as:

(Equation 3) $$\hat{Y}=f_{DNN}\left(X\right)\text{,}$$

(Equation 4) $$Y_{\text{opt}}=\underset{Y}{\text{minimize}}L\left(\hat{Y},Y\right)\text{,}$$

where L is the loss function.

Owing to the considerable diversity inherent in DNNs, we subsequently introduce predominant DNN architectures which can be used in CGH.

#### Network architecture

The universal approximation theorem establishes that DNNs possess theoretically guaranteed capacity to approximate arbitrary continuous functions through learning underlying mathematical relationships.^44^^,^^45^^,^^46^ Nevertheless, reasonable architectural design critically impacts parameter efficiency in practical implementations. As the fundamental architecture of DNNs, the multilayer perceptron (MLP) consists of multiple linear transformation layers:

(Equation 5) Y=Xw+b,

where X is the output of the preceding layer serving as current layer’s input, w denotes the learnable weight connecting neural units, b corresponds to bias, and Y signifies the transformed output. To enable learning of complex functional mappings, nonlinear activation functions are strategically interleaved with linear transformations. The introduction of nonlinear activation functions like Rectified Linear Unit (ReLU)^47^ empowers neural networks to model complex nonlinear relationships essential for solving diverse machine learning problems. Besides standard ReLU and its variants, other seminal activation functions include Sigmoid,^48^ Tanh (hyperbolic tangent) and Softmax,^49^ each offering distinct computational properties for different network architectures.

**Figure 2:**
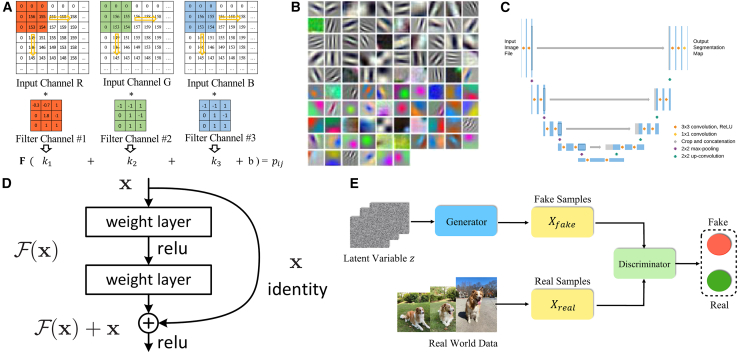
Deep neural network (DNN) architectures used in computer-generated holography (CGH) (A) Convolution operation in color image process and (B) feature map visualization. The convolutional neural network (CNN) applies a set of convolutional filters (also called convolutional kernels) to extract feature maps from the input image and calculate the required results layer by layer.^51^ (C) Basic structure of U-Net. It consists of symmetric downsampling and upsampling blocks to first contract and then expand the image information.^52^ (D) An emblematic building block in residual network (ResNet). ResNet introduces deep residual learning with “shortcut connections”, in which the identity mapping x is added to ℱ(x) to form the output layer, addressing the degradation problem of training accuracy in deeper networks.^53^ (E) Training process for a generative adversarial network (GAN). GAN implements adversarial training with a Generator network and a Discriminator network in competition with each other, where Generator creates forgeries to approach real data while Discriminator distinguishes the real one from the fake one.^54^

Since CGH predominantly operates within image-based frameworks presently, convolutional neural network (CNN) emerges as particularly suitable architectures for processing grid-structured data like digital holograms.^50^ The convolutional layer applies a set of learnable filters (also called kernels) (Figure 2A) to extract hierarchical feature map $Y_{i,j}$ from input images (Figure 2B), which can be mathematically formulated as:

(Equation 6) $$Y_{i,j}=\sum_{m=1}^{M}\sum_{n=1}^{N}w_{m,n}\cdot X_{i-m+1,j-n+1}+b\text{,}$$

where (M,N) stands for the kernel size, enabling successive construction of refined senior feature representations through expanding receptive fields as network depth increases.^51^

Building upon CNN foundations, advanced architectures have been developed for specialized computational tasks. The U-Net architecture,^55^ originally designed for biomedical image segmentation, employs symmetric encoder-decoder structures with skip connections (Figure 2C). The encoder downsamples spatial resolution while extracting contextual features step by step, whereas the decoder reconstructs high-resolution outputs through upsampling operations. Lateral connections between corresponding downsampling-upsampling stages preserve high-frequency details, establishing U-Net as the predominant architecture for CGH applications due to its parameter efficiency and superior reconstruction fidelity.^52^ Another breakthrough architecture, residual network (ResNet), addresses vanishing gradient challenges in deep networks through identity shortcut connections (Figure 2D).^53^ These residual blocks allow gradients to propagate directly through network layers, facilitating effective training of networks exceeding hundreds of layers while maintaining computational stability. Generative adversarial network (GAN) implements a dual-component framework consisting of a generator network that synthesizes data samples and a discriminator network that distinguishes generated samples from real data (Figure 2E).^56^^,^^57^ Once the training is completed, the optimized generator network can autonomously generate novel data samples that achieve distributional congruence with the training set with high fidelity.^54^^,^^58^

Beyond conventional convolutional architectures, emerging vision architectures demonstrate promising potential for CGH.^59^^,^^60^^,^^61^ Vision Transformer (ViT) employs global self-attention mechanisms^10^^,^^62^ to establish long-range spatial dependencies, enabling comprehensive cross-domain mapping from input scenes to holograms. This paradigm shift from local to global processing theoretically enhances reconstruction quality of hologram compared to CNN-based approaches, though at increased computational complexity $O\left(N^{2}\right)$ scaling quadratically with input size.^63^ Addressing this limitation, Vision Mamba (ViM) leverages selective state space mechanism^64^ to achieve linear-time complexity O(N) while maintaining long-range modeling ability. Both ViT and ViM architectures overcome the inherent limitations of CNN’s local receptive fields through global contextual processing, enabling more effective extraction of cross-hierarchical features critical for holographic reconstruction. This architectural evolution suggests new pathways for optimizing the trade-off between reconstruction fidelity and computational efficiency in next-generation CGH systems.

The evolution of DNNs has progressed to an era of architectural hybridization, where CGH increasingly employ integrated frameworks combining complementary network paradigms. Table 1 summarizes key performance characteristics of various DNN architectures for CGH, though optimal selection depends on specific implementation requirements. For example, ResNet-enhanced U-Net integrates residual learning with encoder-decoder structures to achieve high-quality reconstruction while maintaining training stability,^39^ ViT and ViM are also incorporated with CNN to strategically combine global attention mechanisms with local feature extraction, demonstrating improved performance in complex CGH tasks through complementary feature processing.^59^^,^^61^

**Table 1:** Comparison among prevailing DNNs used in CGH

DNN type	Computational efficiency	Reconstruction quality	Memory usage
MLP	High (Simple architecture)	Moderate	Low
U-Net^55^	Moderate (Skip connections increase load)	High (Detail recovery ability)	High
ResNet^53^	Moderate (Shortcuts increase load)	Moderate-High	High
GAN^58^	High (Lightweight architecture)	High (Realism)	Relatively low
ViT^63^	Low (Self-attention brings complexity)	High (Strong global feature extraction ability)	Huge
ViM^65^	Relatively high	High (Long-sequence efficiency)	Relatively low

#### Neural network training and optimization

**Figure 3:**
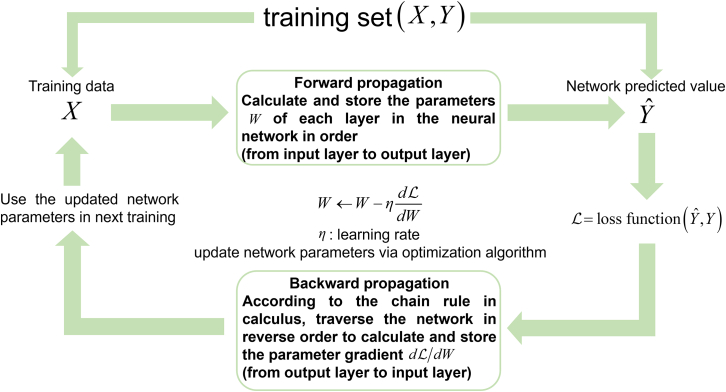
The basic paradigm for training neural networks Generally, a network completes calculation through forward propagation and updates learnable parameters via backpropagation of gradient.

The optimization of neural network parameters is governed by systematic training methodologies, as illustrated in Figure 3. The training process initiates with forward propagation: input data X from the training set is fed into the network, generating predicted value $\hat{Y}$ through successive network layers, while the variable parameters W of each layer are calculated and stored in order (from input layer to output layer). A loss function L quantifies the discrepancy between predictions $\hat{Y}$ and target labels Y. Backpropagation then computes parameter gradients using automatic differentiation based on the chain rule.^66^ Optimization algorithms update parameters with an appropriate learning rate toward minimizing the loss function, and the updated network parameters are used for next training. This iterative process continues until convergence criteria are met, either through reaching maximum epochs or attaining satisfactory loss minimization.

The selection of training methodologies depends fundamentally on data annotation availability, bifurcating into supervised learning (label-dependent) and unsupervised learning (label-independent) paradigms. Supervised approaches minimize the errors between predictions $\hat{Y}$ and annotated labels Y, while unsupervised methods optimize network outputs $\hat{Y}$ directly against input data X.

In addition to datasets, critical design choices include loss function formulation and optimization algorithm. Fortunately, numerous established computational tools exist for these implementations.

##### Loss function design

In CGH applications, virtually all standard image evaluation metrics can be repurposed as loss functions, including mean square error (MSE), peak signal-to-noise ratio (PSNR), structural similarity (SSIM), multiscale structural similarity (MS-SSIM), feature similarity (FSIM), perceptual loss (PL), focal frequency loss (FFL), and so on. The MSE loss function is expressed as:

(Equation 7) $$L_{\text{MSE}}\left(\hat{Y},Y\right)=\frac{1}{N}\sum_{i=1}^{N}{\left({\hat{y}}_{i}-y_{i}\right)}^{2}\text{,}$$

where N is the number of pixels and y represents every pixel value in the image Y. MSE is recognized as one of the most fundamental and extensively employed loss functions in machine learning due to its mathematical simplicity, differentiable nature, and theoretical grounding in minimizing quadratic deviations between predicted values and true values for each pixel. Derived from MSE, PSNR provides a logarithmic measure of reconstruction quality

(Equation 8) $$\text{PSNR}\left(\hat{Y},Y\right)=10\;\log_{10}\left(\frac{R}{L_{\text{MSE}}\left(\hat{Y},Y\right)}\right)\text{,}$$

where R is the maximum value range of pixels in an image. The inverse relationship between MSE and PSNR implies that minimizing MSE directly enhances PSNR values.^67^ However, MSE is sensitive to high-frequency noise and fails to capture structural information, leading to unrealistic images as it overemphasizes pixel-wise errors.^68^ SSIM addresses these limitations in image quality assessment by comparing the structural, brightness and contrast similarity between two images.^69^ The SSIM loss is typically formulated as:

(Equation 9) $$L_{\text{SSIM}}\left(\hat{Y},Y\right)=1-\text{SSIM}\left(\hat{Y},Y\right)=1-\frac{\left(2\mu_{\hat{y}}\mu_{y}+C_{1}\right)\left(2\sigma_{\hat{y}y}+C_{2}\right)}{\left(\mu_{\hat{y}}^{2}+\mu_{y}^{2}+C_{1}\right)\left(\sigma_{\hat{y}}^{2}+\sigma_{y}^{2}+C_{2}\right)}\text{,}$$

where $\mu_{\hat{y}}$ and $\mu_{y}$ are the means of pixel intensity in the predicted and label images, $\sigma_{\hat{y}}$ and $\sigma_{y}$ are the standard deviations, $\sigma_{\hat{y}y}$ is the correlation coefficient between the two images, and two constant $C_{1}$, $C_{2}$ are included to avoid instability when divisor is very close to zero. SSIM focuses on both local and global similarities effectively, providing a holistic index with efficient computation. Afterward, some variant indexes are proposed: MS-SSIM extends SSIM by enhancing multiscale analysis of texture and structure,^70^ and FSIM evaluates feature similarity by comparing the intensity distribution of image features at corresponding locations.^71^ PL utilizes deep learning method to extract deep high-level semantic information and is implemented by minimizing the errors between deep features of generated and true images, leveraging learned representations from a pre-trained DNN to realize better visual perception^72^ but increasing computational burden during training. Differentiating from the above loss functions that compare image features in the spatial domain, FFL considers frequency domain discrepancies in the loss calculation using a dynamic frequency-based weighting, and prioritizes vital hard-to-reconstruct frequency components to reinforce high-frequency detail recovery.^73^ The equation is defined as:

(Equation 10) $$L_{\text{FFL}}=\frac{1}{MN}\sum_{u=1}^{M}\sum_{v=1}^{N}w\left(u,v\right){\left|F_{\hat{y}}\left(u,v\right)-F_{y}\left(u,v\right)\right|}^{2}\text{,}$$

where $w\left(u,v\right)={\left|F_{\hat{y}}\left(u,v\right)-F_{y}\left(u,v\right)\right|}^{\alpha }$ with scaling factor α and $F_{\hat{y}}\left(u,v\right)$, $F_{y}\left(u,v\right)$ is the 2D discrete Fourier transform of predicted and label images.

##### Optimization algorithm analysis

Modern deep learning frameworks provide diverse optimization algorithms: stochastic gradient descent (SGD),^74^ root-mean-square propagation (RMSprop),^75^ adaptive moment estimation (Adam),^76^ each with distinct convergence properties and computational characteristics (summarized in Table 2). SGD is one of the simplest algorithms in terms of computational complexity suitable for small datasets,^43^ while RMSprop introduces adaptive learning rate based on moving average of squared gradients to accelerate convergence. Adam combines momentum (1^st^ moment)^77^ and adaptive learning rate (2^nd^ moment), enabling rapid convergence and performing well in general frameworks.^78^

**Table 2:** Comparison among prevailing optimization algorithms

Optimization algorithms	Convergence speed	Local optima risk	Stability	Memory usage
SGD	Slow (Requires careful tuning)	High	Low (Fixed learning rate causes unstable updates)	Low (Stores only gradients)
RMSprop	Moderate (Adaptive learning rate accelerates convergence)	Moderate	Moderate (Reduced fluctuations)	Moderate (Stores moving average of squared gradients)
Adam	Fast (Combines momentum & adaptive learning rate)	Low	High	High (Stores 1^st^ & 2^nd^ moment estimates)

## Deep learning for CGH

The advent of AI, particularly deep learning frameworks, presents excellent solutions for knotty quality–speed trade-off problems in CGH. As a physically interpretable technology, CGH is certainly applicable to deep learning algorithms to calculate holograms fast and correctly. Based on the different implementation approaches, there are three types: Data-driven models, physics-driven models, and jointly optimized models. These models are innovated with the synergistic development of deep learning and computational optics, which have enabled tremendous success in achieving both high image quality and fast runtime that is not feasible in conventional CGH algorithms.

### Data-driven models

Data-driven models employ supervised learning to establish deterministic mappings between input scenes and target holograms. Given a labeled dataset $\left(I_{\text{tar}},\Phi_{\text{label}}\right)$, where $I_{\text{tar}}$ is the target image and $\Phi_{\text{label}}$ is the label hologram, data-driven models can generate the output hologram $\Phi_{\text{out}}$ as the following formulas:

(Equation 11) $$\Phi_{\text{out}}=f_{\text{model}}\left(I_{\text{tar}}\right)$$

(Equation 12) $$\Phi_{\text{opt}}=\underset{\Phi }{\text{minimize}}L\left(\Phi_{\text{out}},\Phi_{\text{label}}\right)$$

**Figure 4:**
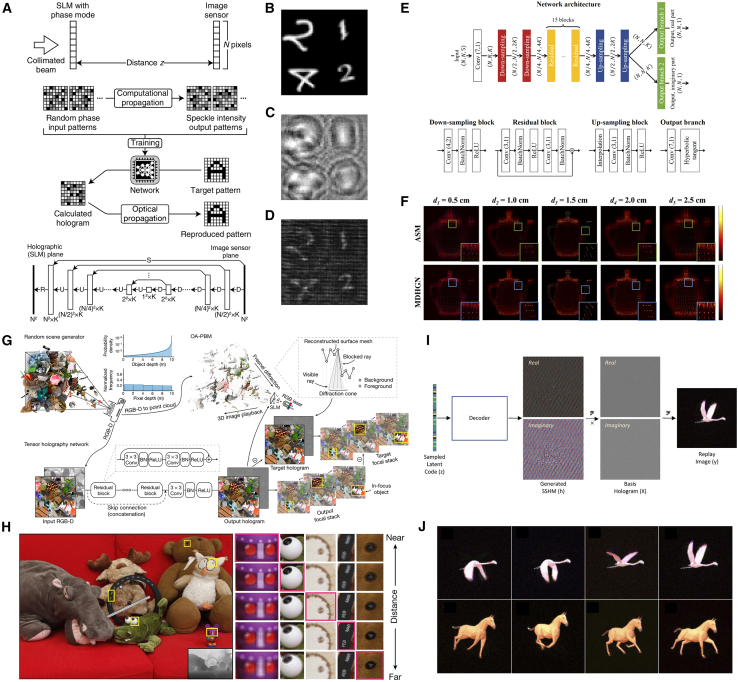
Representative data-driven models (A–D) Deep-learning-generated holography. (A) Method diagram and network design. D: Downsampling block, U: Downsampling block, S: Skip connection, R: Residential block. The trained network takes (B) Target intensity pattern as input and calculates (C) Phase-only hologram (POH). (D) Pattern optically reconstructed from (C).^79^ (E and F) Multi-depth hologram generation network (MDHGN). (E) Architecture of the MDHGN. (F) Comparison in reconstructed images of holograms generated from angular spectrum method (ASM) and MDHGN.^80^ (G and H) Tensor holography V1. (G) Tensor holography V1 workflow. (H) A simulated depth-of-field image and focal stack (the magnified insets) reconstructed from the CNN predicted hologram of an RGBD image.^81^ (I and J) Channeled variational autoencoder (CVAE) model. (I) Generating holograms using the CVAE by feeding latent codes. (J) Replay images of holograms generated by the trained CAVE with different poses.^82^

In 2018, deep learning method was first exploited for CGH,^79^ training a multiscale U-shaped ResNet with 100,000 pairs of random phase patterns and their Fresnel propagating intensity patterns. As shown in Figure 4A, this network learns the inverse diffraction mapping, and experimental validation demonstrated 3.6× acceleration over conventional GS algorithm while maintaining equivalent image quality (Figures 4B–4D). Goi et al. developed a DenseNet^83^ in U-Net architecture to improve parameter efficiency with dense connection and focused on binary hologram generation for efficient holographic display with smaller data volume, alleviating binarization-induced quality degradation in previous methods.^84^

Based on multi-plane holography, Lee et al. proposed the multi-depth hologram generation network (MDHGN) to reconstruct more complicated images.^80^ A pair in the dataset was composed of 5 different images located at various distances and single superposed complex hologram, then the Y-shaped ResNet architecture (Figure 4E) employs dual branches for separate estimation of hologram’s real and imaginary components. To train the network effectively, multiscale patterns (i.e., random sparse and dense dots, circular surfaces) are merged into the dataset, enhancing high-frequency reconstruction fidelity. Comparative results in Figure 4F demonstrates MDHGN’s superiority over conventional angular spectrum method (ASM)^85^ in preserving edge details. Zheng et al. generated 3D POH using similar dual-branch architecture. Their framework achieves equivalent visual quality to 1000-iteration ASM optimization with 50 times reduction in computation time.^86^

Further advancing data-driven models, Khan et al. developed GAN-Holo.^87^ The generator is built upon U-Net to learn the mapping using 64,000 pairs of MNIST images and holograms calculated by Fresnel zone method. Kang et al. innovated point-wise holographic synthesis through a GAN-based framework.^88^ The generator network produces elemental fringe patterns for each object point and then superimposed into an entire hologram like the lookup table (LUT).^89^ Compared to traditional point-based CGH, this approach achieves more than 35 dB PSNR while significantly reducing memory requirements.

The aforementioned results primarily validate the proof-of-concept implementation but lacks full-color reproduction and vivid 3D visual presentation during the exploration stage. In 2021, Shi et al. successfully implemented tensor holography V1 through innovative algorithmic integration, achieving photorealistic full-color 3D holographic display as depicted in Figure 4G and 4H.^81^ They established a pioneering large-scale CGH dataset (MIT-CGH-4K-V1) by generating 4,000 RGB-depth (RGBD) image and hologram pairs, employing an occlusion-aware point-based method (OA-PBM) to improve 3D hologram generation accuracy. A fully convolutional residual network (architectural details in Figure 4G), featuring a lightweight design, was optimized to produce complex holograms, subsequently encoded into POHs precisely using an anti-aliasing double-phase modulation (AA-DPM). This framework is a milestone breakthrough in DLCGH, demonstrating the synergistic potential of deep learning and holography through real-time processing capabilities on commercial GPUs or even mobile phones and exceptional image fidelity at full high definition (FHD) resolution (1920 × 1080), thereby establishing critical infrastructure for holographic display commercialization.

Inspired by this notable progress, subsequent research efforts have increasingly focused on stereoscopic holography. Liu et al. designed the channeled variational autoencoder (CVAE) model that introduces additional latent variables assigned to specific color channels, enabling a unified network to generate full RGB holograms through different latent code inputs (Figure 4I).^82^ Their methodology innovatively incorporated spatial spectrums of hologram modulator (SSHMs) as functional relationships between the basis and target holograms rather than holograms themselves as training data, substantially enhancing the network’s generalization capacity for generating new holograms with varied stereoscopic poses (Figure 4J). CHoloNet demonstrated multi-plane/multi-wavelength hologram generation with no color crosstalk.^90^ Yang et al. advanced the field through diffraction-engineered holography (DEH), constructing an ASM-based RGBD dataset with physically accurate varifocal renderings to train DEHNet similar to tensor holography V1, achieving photorealistic reconstruction (33.4 dB PSNR, 0.957 SSIM) with natural defocus effects at FHD resolution.^91^ The framework evolution continued with Chang et al.’s end-to-end stereo-to-hologram network (SHNet), utilizing binocular image pairs to synthesize view-based 3D complex holograms as training dataset.^92^ Their subsequent research integrated monocular depth estimation via MiDaS^93^ to create hybrid 2D-3D training pairs, enabling direct hologram generation from single-view 2D inputs without explicit 3D conversion.^94^

Table 3 summarizes representative data-driven models and associated evaluation metrics. The preceding analysis reveals that creating an elaborate dataset containing holograms before training has been a top priority for these data-driven models, which remains a considerable challenge for everyone. Instead of innovations in network design, data-driven models pay more attention to the acquisition and establishment of labeled datasets. On the one hand, as a record of certain optical information, hologram is so unique that we must recalculate it once we change any conditions like propagating distance, wavelength, pixel size or resolution, etc., leading to a dilemma that one cannot use existing hologram datasets directly to handle new problem. On the other hand, we should pursuit the improvement of the hologram fidelity to offer ever better image resolution free of artifacts. With recent advancements in computational infrastructure and artificial intelligence capabilities,^95^ we anticipate accelerated development of high-resolution and high-fidelity hologram datasets through synergistic integration of physical modeling and machine learning techniques.

**Table 3:** Summary of representative data-driven models

Data-driven models	DNN type	Resolution	PSNR (dB)	SSIM	Calculation time (ms)	3D support	Full-color demonstration
Deep-learning-generated holography^79^	U-Net + ResNet	64 × 64	/	/	26	✕	✕
MDHGN^80^	ResNet	512 × 512	>19	/	46.8 per depth	✓	✕
GAN-based CGH^88^	GAN	16 × 16 32 × 32	44.56 35.11	/	/	✕	✕
Tensor Holography V1^81^	ResNet	1920 × 1080	∼34 (Focal stack)	∼0.97 (Focal stack)	40	✓	✓
CVAE^82^	Variational autoencoder	128 × 128	/	/	<5	✓	✓
DEHNet^91^	ResNet	1920 × 1080	33.4	0.957	16	✓	✓
From picture to 3D hologram^94^	U-Net + ResNet	1024 × 512	∼23	/	17.5	✓	✕

“/” means no related data in the references. “∼” means estimated values from the references.

### Physics-driven models

The core objective in developing hologram-generating neural networks focuses on enhancing the reconstruction fidelity of holographic display. However, CGH fundamentally constitutes an ill-posed inverse problem where deriving precise analytical solutions remains challenging.^96^ This reveals an inherent paradox in data-driven models: while demanding superior performance over traditional CGH methods, such models depend on pre-established hologram datasets through those conventional techniques. Consequently, the quality of training hologram labels inherently defines the performance ceiling for network-generated holograms. To transcend these limitations, recent advances leverage differentiable physical simulations of light propagation (Fraunhofer diffraction, Fresnel diffraction, and ASM propagation) within deep learning frameworks. These differentiable algorithms maintain computational gradients throughout numerical processes which are precisely the values required for neural network optimization. This synergy enables physics-driven models that bypass dependency on labeled datasets by directly optimizing holograms through end-to-end differentiable pipelines. Specifically, these models numerically propagate generated holograms to target planes and compute loss functions between reconstructed and ground-truth scenes, thereby aligning optimization objectives with perceptual quality metrics, which is more closely with the intention of sufficiently utilizing deep learning. This approach can be described as:

(Equation 13) $$I_{\text{pred}}=f_{\text{prop}}\left(\Phi_{\text{out}}\right)=f_{\text{prop}}\left(f_{\text{model}}\left(I_{\text{tar}}\right)\right)\text{,}$$

(Equation 14) $$\Phi_{\text{opt}}=\underset{\Phi }{\text{minimize}}L\left(I_{\text{pred}},I_{\text{tar}}\right)\text{,}$$

where $f_{\text{prop}}$ is the simulated light propagation process and $I_{\text{pred}}$ is the prediction of reconstructed image from hologram, so that the dataset only needs to provide the original target image $I_{\text{tar}}$ without label holograms.

**Figure 5:**
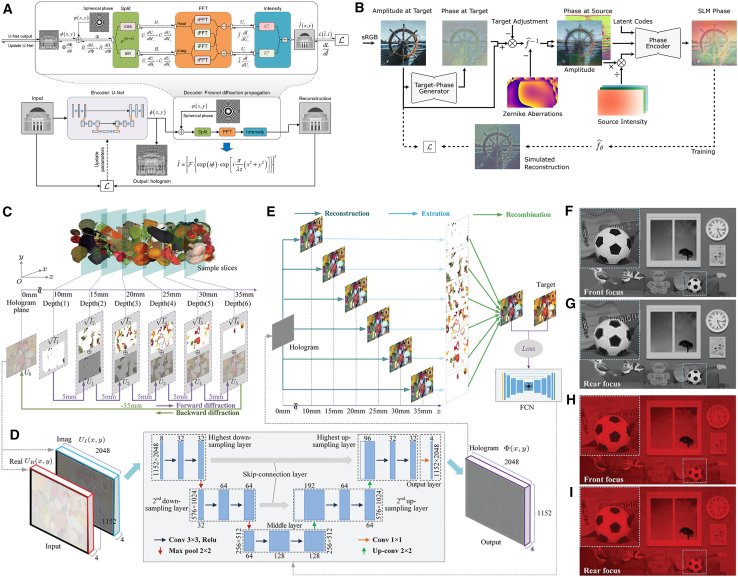
Representative physics-driven models (A) Architecture of Holoencoder. U-Net, as the encoder, generates hologram from input target image, and Fresnel diffraction propagation model, as the decoder, propagates the hologram to output reconstructed image. The reconstructed image is compared with target image directly to calculate loss and update learnable parameters in encoder network without label hologram during training, which is called unsupervised learning.^97^ (B) HoloNet workflow. The 1st subnetwork (Target-Phase Generator) calculates the phase information of the target field and the 2nd subnetwork (Phase Encoder) encodes the amplitude and phase of the SLM field into POH. Target field and SLM field are connected by light propagation algorithm (Zernike-compensated propagation operator) to form the physics-driven model.^98^ (C–I) Multi-depth 3D hologram. (C) The computation of the multi-depth diffraction field through forward-backward-diffraction framework. (D) The structure of the network. (E) The calculation of multi-depth loss while training. (F-I) The numerical reconstruction (F and G) and optical reconstruction (H and I) of front focus plane and the rear focus plane.^99^

Holoencoder implemented unsupervised learning in CGH, employing a U-Net encoder coupled with a Fresnel diffraction decoder to directly synthesize 4K (3840 × 2160) POHs within 150 ms.^97^ A critical innovation involved decoupling complex amplitude computation using Euler’s formula (Figure 5A), enabling accurate gradient backpropagation through the physical model. Afterward, 4K diffraction model-driven network (4K-DMDNet) integrated sub-pixel convolutional layers with oversampled diffraction calculations, achieving 20.49 dB average PSNR on test sets for 4K POH generation within 260 ms.^100^ Wang et al. established physical model-driven network (PMD-Net) with band-limited angular spectrum diffraction algorithm and unsharp filter enhancement to suppress reconstruction artifacts.^101^ Alternative unsupervised approaches demonstrate wavefront superposition via U-Net-predicted phase distributions to compose the POH,^102^ further expanding design paradigms in CGH.

The abovementioned physics-driven models only employ unidirectional light propagation in the hologram generation pipeline, rendering them susceptible to reconstruction distance and scalability limitations. A significant advancement emerged in 2020 with DeepCGH implementing Fourier holography principles.^103^ This framework involves four processes: (1) employing CNN to transform multi-channel 3D target amplitude distributions into complex field representations at the target plane, (2) deriving POHs at the SLM plane through inverse 2D Fourier transforms, (3) propagating these POHs across varying distances to match the input planes, and (4) calculating loss between results and target amplitude patterns. Peng et al. developed HoloNet with a dual-subnetwork architecture capable of real-time generation for FHD POHs.^98^ As illustrated in Figure 5B, this workflow bifurcates the hologram generation into two steps: (1) Target-phase generator subnetwork processes target amplitude input to produce target phase distributions, and the predicted phase and original amplitude are then propagated forward to the SLM plane to form complex field representations. (2) phase encoder subnetwork transforms the SLM complex field to final POH, which is backpropagated to reconstruct the simulated amplitude and compare with target amplitude. The process can be expressed using a formula as:

(Equation 15) $$I_{\text{pred}}=f_{\text{prop}-}\left(\Phi_{\text{out}}\right)=f_{\text{prop}-}\left(f_{\text{model}}\left(I_{\text{tar}}\right)\right)=f_{\text{prop}-}\left(f_{\text{DNN}2}\left(f_{\text{prop}+}\left(f_{\text{DNN}1}\left(I_{\text{tar}}\right)\right)\right)\right)\text{.}$$

Crucially, this forward and backward propagation framework fundamentally decouples network training from specific propagating distances. This design constraint forces the model to prioritize scene feature extraction over learning physical propagation, as the diffraction distance is solely determined by the predefined simulation parameters. Inspired by this framework, physics-driven models have undergone substantial exploration, yielding significant advancements encompassing network architecture optimization, training strategy refinement, 3D visual enhancement, and differentiable propagation algorithm development.

Some teams innovated network architectures to advance DLCGH. Phase dual-resolution network (PDRNet) employed a dual-branch architecture for multiscale context extraction.^104^ Through strategic implementation of group convolution, this approach achieved high-fidelity FHD holograms in 57 ms with 31.17 dB PSNR and 0.93 SSIM while reducing model complexity. Dong et al. introduced a Fourier based operator compatible with conventional CGH architectures, systematically integrating forward/inverse Fourier transforms around convolution layers to enhance global feature extraction and substantially improve reconstruction quality.^105^ Considering the amplitude and phase characteristics of optics, Zhong et al. developed a fully complex-valued CNN architecture specifically designed to model complex field.^106^ Recent architectural innovations include a hybrid asymmetrical neural network combining real-valued convolutions in phase generation with complex-valued operations in phase encoding, augmented by Fourier transforms, achieving 34.98 dB PSNR and 0.95 SSIM at 20 ms for FHD holograms.^107^ Stemmed from DPAC, dual-channel parallel neural network (DCPNet) was proposed to predict two sub-POHs followed by checkerboard sampling to synthesize a single FHD POH in 36 ms with 31.31 dB PSNR and 0.86 SSIM.^108^ The multilevel wavelet-based channel attention network (MW-CANet) employed discrete wavelet transform (DWT) decomposition for multi-frequency feature learning, producing FHD POHs in 24 ms with 34.81 dB PSNR and 0.918 SSIM.^109^ Qin et al. proposed complex-valued generative adversarial network (CV-GAN) to process complex-valued data and compare the amplitude of the reconstructed image with the target amplitude via the discriminator, further enhancing the fitting ability through adversarial learning.^110^

Some groups have dedicated substantial efforts to develop advanced training methods. Wang et al. trained a U-Net employing low-frequency mixed noise instead of conventional real-image inputs, achieving 29.2 dB PSNR.^111^ Zhu et al. engineered a specialized training dataset comprising orthogonal Fourier basis functions, significantly enhancing spectral representation fidelity and attaining 31.16 dB PSNR with 0.942 SSIM at FHD resolution.^112^ Res-Holo integrated a pretrained ResNet34 encoder with an adaptive focal frequency loss mechanism, optimizing frequency-domain feature extraction to generate FHD POHs at 14 ms with 32.88 dB PSNR.^39^ Zhong et al. encoded POH through a learnable layered initial phase to replace the first phase-generator subnetwork, achieving 36.87 dB PSNR and 0.96 SSIM in 4K resolution.^113^ Yan et al. systematically investigated CNN-based double-phase encoding techniques, producing 4096 × 2400 POHs with 35.6 dB PSNR in 0.06 s while effectively suppressing fringe artifacts and spatial shifting noises.^114^ Cross-domain fusion network (CDFN) was presented with multi-stage deep supervision mechanism, enforcing the network to learn from early initial-stage feature maps to optimize amplitude-to-phase transformations, generating POHs with 31.68 dB PSNR and 0.944 SSIM within 12 ms.^115^ Load sharing yielding holography (LSY-Holo) leveraged the novel Global SSIM as loss function to train a Y-shaped dual-branch architecture network and yield POHs of binary images with 26.71 dB PSNR and 0.7412 SSIM.^116^

Significant achievements have been achieved in developing authentic 3D holographic reconstruction. Self-holo was proposed for 3D POH^117^ by employing RGBD image inputs to represent 3D objects. The generated hologram was randomly propagated to one of the sliced layers, thereby eliminating computational dependency on layer numbers and optimizing resource utilization. Song et al. conceived a modified 30-layer-depth training model enabling real-time holographic display at 22 fps.^118^ Wang et al. achieved full-color holography with an end-to-end physical model-driven network (EEPMD-Net) that simultaneously synthesizes RGB 3D POHs in FHD resolution within 0.15 s, achieving a PSNR of ∼28 dB while reducing computational demands.^119^ Yan et al. introduced an occlusion-enhanced forward-backward-diffraction framework capable of producing 3D holograms (Figures 5C–5E). Their approach utilized a curated 4K-resolution RGBD dataset comprising 800 training and 100 testing images. After the multi-depth diffraction field of the RGBD dataset has been computed via layer-by-layer replacement method, the complex amplitude diffraction fields was decomposed into real and imaginary part as network input explicitly, generating 4K POH in 90 ms with precise depth focusing (Figures 5F–5I).^99^ Notably, their subsequent investigation demonstrated that neural network’s encoding capacity depends primarily on distributions of intensity and depth rather than their correspondence, employing a random mapping + resampling method of 2D datasets to produce virtual depth values, creating synthetic RGBD training data that yielded excellent 3D focusing in 4K POH reconstruction.^120^

Several research groups concentrate on ameliorating physical propagation algorithms. Ishii et al. enhanced the DLCGH framework^121^ with aliasing-reduced scaled and shifted (ARSS) Fresnel diffraction computation,^122^ where a DNN generates POHs enabling reconstructed images exceeding original sizes. Addressing zeroth-order diffraction disturbance of unmodulated light, Liu et al. developed a U-Net architecture incorporating mimetic phase gratings, achieving 4K POH generation in the first diffraction order within 0.05 s with 37.2dB PSNR and 0.99 SSIM.^123^

**Figure 6:**
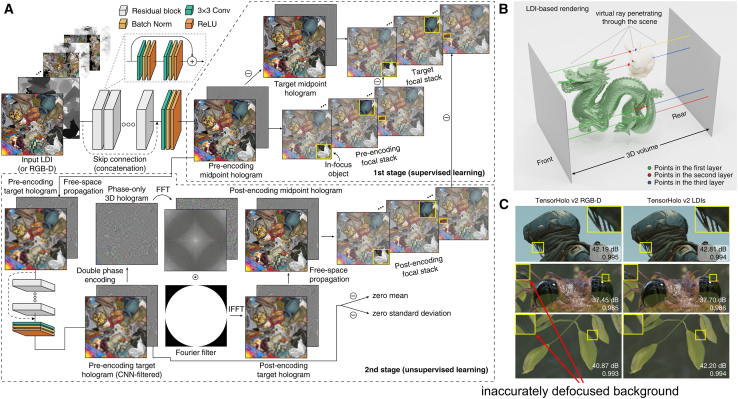
Tensor holography V2^124^ (A) Training procedure of supervised + unsupervised learning. The first net predicts the midpoint holograms compared with supervised labels, and the second net searches for the best pre-encoding complex holograms with unsupervised optimization. (B) Rendering of a layered depth image (LDI) records the intersections between a penetrating ray (colored differently after every new hit) and the scene objects (front-facing surfaces) at every spatial location. (C) LDI-based rendering eliminates inaccurately defocused background compared with RGBD images.

Moreover, emerging evidence demonstrates that hybrid supervised+unsupervised learning frameworks surpass individual approaches in performance. Tensor holography V2 first introduced two-stage training protocol,^124^ where initial supervised CNN training predicts midpoint complex holograms using ground truth labels, followed by unsupervised second CNN that discovers optimal pre-encoding complex holograms (Figure 6A). Leveraging the MIT-CGH-4K-V2 dataset, which employs layered depth images (LDI, Figure 6B) as computationally efficient 3D representations, this framework achieved photorealistic reconstruction metrics (PSNR: 29.1 dB, SSIM: 0.944). Comparative analysis in Figure 6C confirms LDI’s superiority over traditional RGBD formats. Subsequent studies have adopted analogous semi-supervised paradigms, successfully enhancing 3D depth perception in holography.^125^^,^^126^^,^^127^^,^^128^

Table 4 summarizes representative physics-driven models and their relevant evaluation metrics. These models have realized the full potential of deep learning by integrating light propagation priors, offering three principal advantages. First, their compatibility with existing public datasets, particularly computer vision repositories,^129^^,^^130^^,^^131^ eliminates the need for model-specific label hologram dataset development. Second, their fundamental mechanism transcends label hologram limitations through physical inverse problem solving rather than direct mapping approximation, ensuring superior reconstruction quality. Third, the framework maintains operational flexibility for customized training under varied physical constraints. However, the incorporation of physical equations into neural network architectures incurs significant computational overhead and reduced processing efficiency, due to the iterative execution of light propagation algorithms during training cycles, necessitating the development of advanced computing hardware to mitigate these constraints.^132^ More fundamentally, current implementations predominantly employ 2D planar light propagation algorithms, restricting holographic display to fixed-view 2D slices. While RGBD/LDI-based 3D CGH extends capabilities through z axis layered processing, these calculations remain confined to 2D image planes rather than true volumetric space, preventing us from displaying holographic scenes with multiple perspectives and directions.

**Table 4:** Summary of representative physics-driven models

Physics-driven models	DNN type	Resolution	PSNR (dB)	SSIM	Calculation time (ms)	3D support	Full-color demonstration
**Unidirectional propagation**							

Holoencoder^97^	U-Net	3840 × 2160	23.2	/	150	✕	✕
PMD-Net^101^	U-Net + ResNet	1920 × 1072	/	∼0.9	∼50	✓	✕
4K-DMDNet^100^	U-Net + ResNet	3840 × 2160	20.49	/	260	✓	✓

**Forward & backward propagation**							

DeepCGH^103^	U-Net	1024 × 1024	/	/	8.7	✓	✕
HoloNet^98^	U-Net	1920 × 1072	∼30	/	∼25	✓	✓
Tensor Holography V2^124^	ResNet Improved on the basis of v1	1920 × 1080	29.6	0.947	16	✓	✓
Fourier inspired module^105^	w/HoloNet	1920 × 1072	33.508	0.961	20	✕	✕
Res-Holo^39^	w/pretrained ResNet34	1920 × 1072	32.88	0.95	14	✕	✓
CV-GAN^110^	GAN w/U-Net	1920 × 1072	33.68	0.9526	∼19	✕	✕
EEPMD-Net^119^	U-Net + ResNet	1920 × 1072	∼28	/	≤53	✓	✓
Multi-depth 3D holograms^99^	U-Net	3840 × 2160	31.8	0.86	90	✓	✕
CDFN^115^	U-Net	1920 × 1072	31.68	0.944	12	✕	✓

“/” means no related data in the references. “∼” means estimated values from the references.

### Jointly optimized models

Although deep learning has brought new vitality to generating holograms, one can never neglect that the purpose we devote into CGH ultimately is aimed at practical implementations spanning display technologies, advanced imaging systems, and cross-disciplinary scientific applications. Merely considering the generation of hologram is clearly not enough and we must go further to address real-world operational challenges through intelligent system integration. This imperative drives the emergence of jointly optimized models that synergizes DNNs with optical platforms like metasurface and systematically compensate optical aberrations arising from real-world experimental conditions. This paradigm can be broadly described as:

(Equation 16) $$I_{\text{real}-\text{world}}=f_{\text{real}-\text{world}-\text{prop}}\left(\Phi_{\text{out}}\right)=f_{\text{real}-\text{world}-\text{prop}}\left(f_{\text{model}}\left(I_{\text{tar}}\right)\right)$$

(Equation 17) $$\Phi_{\text{opt}}=\underset{\Phi }{\text{minimize}}L\left(I_{\text{real}-\text{world}},I_{\text{tar}}\right)\text{,}$$

where $f_{\text{real}-\text{world}-\text{prop}}$ is not perfect simulation but considers concrete errors brought by the imperfections in the real world.

**Figure 7:**
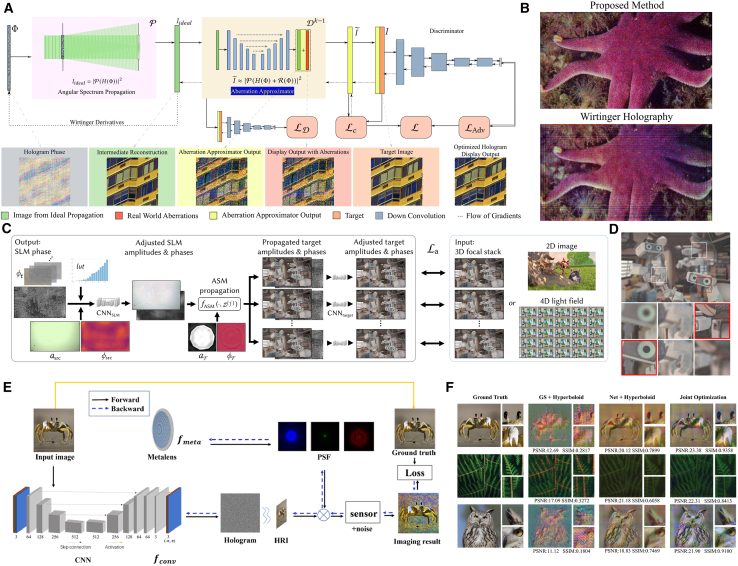
Representative jointly optimized models (A and B) Hardware-in-the-loop phase retrieval method. (A) Overall pipeline. Real world errors in holographic image reconstruction are compensated by a trained neural network that acts as a differentiable surrogate model from the ideal simulated reconstruction to the aberrated real world display. (B) Image captured on real hardware that is aberration-free and closer to the target image compared with advanced Wirtinger holography.^133^ (C and D) Time-multiplexed neural holography. (C) Illustration of calibrated wave propagation model and 2D/3D/4D training strategy. CNN_SLM_ adjusts SLM complex amplitude field and CNN_target_ calibrates the propagated wave field at every target plane separately. (D) 3D CGH reconstruction results captured by a display prototype with natural out-of-focus blur.^134^ (E and F) Holographic meta-based imaging system. (E) Schematic diagram. The trainable parameters of network and the phase profile of metalens are jointly optimized based on point spread function (PSF) simulation and imaging process. (F) The imaging results of proposed joint optimizing methods and comparison with GS and network.^135^

Almost all previously mentioned DLCGH solutions employ idealized coherent light propagation models and neglect practical deviations from optical component imperfections and environmental perturbations, often leading to terrific simulation results but deteriorated practical experiment performances. To bridge this discrepancy, Chakravarthula et al. developed a learned hardware-in-the-loop optimization method that compensates for aberrations between simulated reconstruction and real-world display.^133^ Their architecture integrates a GAN-based aberration approximator (Figure 7A) into phase retrieval pipeline, demonstrating reconstruction improvements of >10 dB PSNR in simulation and >2.5 dB in hardware validation (Figure 7B). Neural holography with camera-in-the-loop (CITL) learning strategy incorporates a Zernike polynomial-parameterized wave propagation model to emulate complex hardware configurations.^98^ This technique achieved 19.5 dB PSNR and 0.6 SSIM on real-world captured images, reducing severe noise artifacts of existing holographic display. Afterward, Choi et al. introduced neural 3D holography with an accurate plane-to-multiplane network-parameterized model based on CNNs to realize 3D holographic VR/AR display in FHD resolution.^136^ Time-multiplexed neural holography was also demonstrated on the basis of neural 3D holography to realize CGH joint optimization. As shown in Figure 7C, this framework incorporates two key components: CNN_SLM_ for SLM optical field correction and CNN_target_ for optimizing propagated fields across multiple focal planes. Notably, the wave propagation model integrates learned optical filters with wavelength-specific aperture sizes for RGB color channels. As a versatile framework supporting different types of input content, including 2D and RGBD images, 3D focal stacks, and 4D light fields, Time-multiplexed neural holography achieved pixel-level depth clues between time-multiplexed frames (Figure 7D).^134^ Xia et al. addressed hardware-induced artifacts through their SGD-Unet architecture, successfully reconstructing images under non-uniform illumination conditions.^137^ To combat inherent limitations in diffraction propagation methods that produce ringing artifacts in practical holographic displays, Yuan et al. developed a diffraction propagation error-compensation network. Integrated with HoloNet, this solution achieved 32.47 dB PSNR in simulated quantitative results.^138^

The characteristics of light source play a crucial role in CGH hardware systems. Owing to the eye safety and speckle artifacts associated with highly coherent laser sources in wavefront modulation,^139^ a partially coherent light source propagation model for light-emitting diodes (LEDs) and superluminescent LEDs (SLEDs) was evaluated to achieve speckle-reduced, high-contrast holography.^140^ Tong et al. further analyzed spatial coherence effects on holographic imaging quality, implementing a U-shaped residual dense network (U-RDN) to recover and improve the scenario information of low-coherence CGH.^141^ Besides, multi-color holograms with three intensity-modulated light sources were optimized through MLP network, enabling precise laser power control per subframe to enhance brightness while resolving the visual distortions and color mismatches.^142^ A deep learning-enabled camera system DeepIHC combined real-world acquisition hardware with hologram filtering neural network to diminish noise and enhance the visual quality of incoherent holograms.^143^

For the optical reconstruction of CGH, traditional methods primarily employ SLM or DMD to load holograms. These devices exhibit high manufacturing complexity and cost constraints, particularly due to their bulky pixel sizes that restrict precise light modulation. Differentiating from conventional optical modulation devices, metasurface, an engineered 2D platform featuring subwavelength-scale unit structures, demonstrates peculiar electromagnetic response properties unattainable in natural materials. Capable of manipulating phase transitions within optical wavelengths^144^ and achieving large diffraction angles,^33^ metasurface provides novel solutions for holographic display with broad field of view. This potential has driven research initiatives replacing traditional optical components with metasurface. Yu et al. proposed a metalens-based compact miniaturized full-color holographic imaging system, where the trainable parameters of network and the phase profile of metalens are jointly optimized based on point spread function (PSF) simulation and imaging process, as depicted in Figure 7E, achieving quite higher imaging quality of more than 3 dB PSNR and 0.2 SSIM in contrast to network without joint optimization (Figure 7F).^135^ Building on the established DeepCGH framework,^103^ Xi et al. implemented metasurface inverse design for polarization-multiplexed holography, obtaining different patterns with maximally four co- and cross-polarization conversion channels.^145^

**Figure 8:**
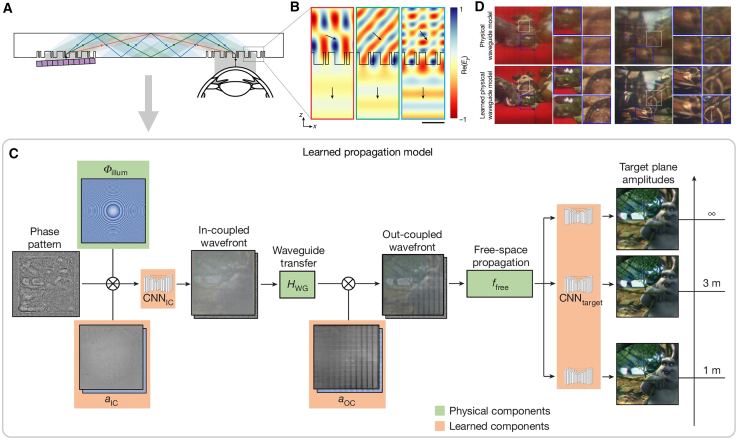
Full-color 3D holographic AR glasses with metasurface waveguides^146^ (A) Visualization of the waveguide geometry for full-color operation. This prototype uses high-index glass metasurface gratings that are optimized for maximum diffraction efficiency and uniformity of angular response to transmit all red, green and blue wavelengths. (B) Electric field maps at red (638 nm), green (521 nm) and blue (445 nm) wavelengths for light passing through the metasurface out-coupler toward the user’s eye. (C) Illustration of the learned waveguide propagation model. This model combines physical aspects of the waveguide (highlighted in green) with AI components that are learned from camera feedback (highlighted in orange) to accurately mimic physical optics in practice. (D) Comparison of captured 3D experimental results using physical model and proposed learned physical model.

Notably, Gopakumar et al. developed a breakthrough full-color 3D holographic AR glasses prototype,^146^ which conspicuously epitomizes the joint optimization method. As depicted in Figure 8, this work combines a dispersion-compensating waveguide (Figure 8A), inverse-designed metasurface gratings (Figure 8B), and deep-learning–driven algorithms (Figure 8C), which are co-designed to eliminate the need for bulky collimation optics between the SLM and the waveguide, achieving unprecedented compactness and visual fidelity (Figure 8D) that bridges theoretical innovation with practical commercialization potential.

We systematically categorize representative jointly optimized models and their performance metrics in Table 5. Compared with physics-driven models, jointly optimized models not only incorporate ideal theoretical conditions but also address practical implementation challenges involving optical components, aberrations, and distortions. Through synergistic combination of software algorithms and hardware configurations, they achieve enhanced visual perception while maintaining system compactness. In this practical application-oriented workflow, the optimization paradigm shifts from simulation metrics to the perceptual quality of hardware-implemented holographic scenes, prioritizing end-user visual experience through physical display prototypes. These models employ adaptive calibration mechanisms that transform intractable propagation errors into learnable network parameters via self-adjusting optical field modulation, thereby establishing a robust framework for aberration correction. However, joint optimization with the comprehensive consideration of practical constraints typically sacrifices computational efficiency, necessitating further investigation into real-time implementation strategies.

**Table 5:** Summary of representative jointly optimized models

Jointly optimized models	DNN type	Resolution	PSNR (dB)	SSIM	Calculation time (ms)	3D support	Full-color demonstration
**Holographic displays**							

Learned hardware-in-the-loop^133^	GAN	1920 × 1080	20.5^a^	0.625^a^	/(iterative)	✕	✓
Camera-in-the-loop (CITL)^98^	MLP + CNN	1920 × 1072	19.5^a^	0.60^a^	/(iterative)	✓	✓
CGH w/partially coherent light^140^	w/CITL	1920 × 1080	22.4^a^	/	/(iterative)	✓	✓
Neural 3D holography^136^	CNN	1920 × 1080	22.7^a^	0.79^a^	/(iterative)	✓	✓
Time-multiplexed neural holography^134^	CNN	1920 × 1080	22.85^a^	0.770^a^	/(iterative)	✓	✓
DeepIHC^143^	ResNet	1024 × 1024	∼19.95^a^	∼0.74^a^	47.6	✓	✓
Error-compensation network^138^	U-Net w/HoloNet	1920 × 1072	32.47	0.904	63	✕	✕

**Holography w/metasurface**							

Metalens-based holographic imaging system^135^	U-Net	1080 × 1080	20.81	0.8423	/	✕	✓
Deep-Learning Assisted Polarization Holograms^145^	MLP w/DeepCGH	64 × 64	/	/	40 times faster than GS	✕	✕
Full-color 3D holographic AR glasses w/metasurface waveguides^146^	CNN	/	∼23.32^a^	/	several minutes	✓	✓

“/” means no related data in the references. “∼” means estimated values from the references.

a Note that PSNR and SSIM in this cell are obtained not from numerical simulation results like previously mentioned models but from actually captured images in real-world experiments.

## Conclusion

In summary, AI especially deep learning has significantly enriched the vibrancy of CGH across multiple dimensions. From a developmental standpoint, DLCGH has achieved numerous advancements through continuous exploration: transitioning from grayscale to full-color imaging, low-resolution to high-resolution outputs, and 2D representations to 3D visualizations. These advances have enabled DLCGH to not only catch up with traditional CGH algorithms but also surpass their performance benchmarks, positioning it as a promising candidate for practical holographic display implementations. Differentiating from conventional CGH algorithms, DLCGH demonstrates unparalleled advantages through its operational paradigm: once a network model undergoes training and parameter optimization, it can generate new holograms with exceptional generalization capabilities. This innovative approach eliminates the necessity for repeated optimization processes when adapting to new target scenes.

Table 6 systematically evaluates three predominant DLCGH models. Data-driven models require labeled hologram datasets for training networks through supervised learning, with the critical factor revolving around precise label hologram generation rather than framework complexity. While these models exhibit superior computational efficiency compared to alternatives, their reconstruction fidelity remains constrained by inherent defects of pre-computed holograms in training data. Physics-driven models integrate DNNs with physics-based simulations, employing unsupervised learning to minimize discrepancies directly between reconstructed images and target images through loss function optimization. These models ensure image quality aligns with physical principles, overcoming limitations imposed by purely data-driven approaches. Jointly optimized models, as their name implies, comprehensively address practical implementation challenges by balancing numerical simulation accuracy with real-world environmental factors, requiring significant computational investments. Such methods sometimes employ paired simulated-image and experimentally captured real-world image datasets to mimic system mismatches,^98^^,^^133^ or frequently utilizing multi-stage neural network architectures and iterative optimization procedures^134^^,^^136^^,^^146^ that result in the difficulty of achieving real-time computation.

**Table 6:** Comparison among three DLCGH methods

DLCGH methods	Require labeled dataset	Real-time generation	Real-world image quality
Data-Driven Models	Yes	Easy	Relatively low
Physics-Driven Models	No	Relatively easy	Better
Jointly Optimized Models	Sometimes yes	Hard	Best

Besides, recent advancements in DLCGH highlight its growing synergy with diverse research domains. This is exemplified by successful implementation of image super-resolution in CGH frameworks,^127^^,^^147^ where spatial resolution-enhanced holograms are produced from low-resolution RGBD images, and of optimized image compression architectures,^148^^,^^149^^,^^150^ which leverage neural network encoding to reduce data redundancy of holograms while preserving perceptual quality. Further expanding its utility, noise-suppression pipeline has been integrated into DLCGH,^151^ employing denoise convolutional neural network (DnCNN) to remove speckle noise in hologram reconstruction. These abovementioned works demonstrate deep learning techniques from other fields can also be utilized to help DLCGH achieve better image quality and solve specific problems such as data transmission and compression.

## Challenges and perspectives

This paper systematically reviews and categorizes the current state-of-the-art DLCGH methods, categorizing methodologies into data-driven, physics-driven, and jointly optimized models. It underscores the transformative potential of DLCGH in overcoming traditional CGH limitations, such as computational inefficiency and reconstruction artifacts, through neural network innovations and physical modeling. Key advancements include real-time hologram generation, high-fidelity 3D reconstruction, and hardware-software co-design for practical applications like AR/VR displays. However, the rapid evolution of DLCGH has unveiled both transformative opportunities and critical challenges. To advance the field toward practical, high-fidelity applications, we propose three pivotal research trajectories that may require further exploration and investigation.

### Physics-aware network design with interpretability and flexibility

Current DLCGH architectures predominantly rely on established computer vision paradigms, translating optical information into conventional visual representations to address specific imaging challenges. Although CNN, ViT, and ViM have found widespread application in computational optics, their treatment as “**black boxes**” overlooks the unique physical properties inherent in optical fields (encompassing amplitude, phase, polarization, and other properties). In other words, these networks lack of interpretability. Future architectures may explicitly model physical wavefront propagation dynamics, such as complex-valued CNNs^106^^,^^110^ and physics-aware network mechanisms, to preserve optical fidelity while reducing computational overhead.

Additionally, most current CGH networks require retraining when input parameters change (wavelength, propagation distance, or hardware configurations), leading to a lack of flexibility in adapting to diverse operational conditions. Solutions to this challenge include model-based plug-and-play solvers that integrate traditional iterative algorithms with neural networks to enable adaptive parameter tuning without retraining.^152^^,^^153^ Another promising approach is flexible network module design. For example, convolutional position embedding (CPE) module enhances model flexibility by encoding spatial relationships invariant to input size.^154^^,^^155^ By leveraging CPE, it may possible to generate high-quality, large-scale holograms even when training with small-resolution images.

### Efficient and lightweight network for real-time CGH generation

To fully capitalize on the advantages of CGH display, real-time hologram generation and reconstruction remain imperative. Despite significant advancements over traditional CGH optimization methods, current DLCGH models are still constrained by computational limitations, resulting in insufficient frame rates (10–60 Hz) and spatial resolution levels (typically 1080p or lower) which substantially lag behind industry-standard display specifications (120 Hz refresh rates and 2K/4K resolution). This performance gap not only restricts practical commercialization potential but also highlights critical technical challenges in achieving photorealistic and high-speed holographic display. Furthermore, in many jointly optimized models, deep learning currently serves only a supplementary role since primary optimization remains reliant on conventional iterative algorithms.^146^^,^^156^ There is a critical need for DLCGH models that integrate DNNs as much as possible throughout the entire optimization pipeline to improve the computing speed while guaranteeing the image quality. To address these challenges, various strategies can be employed.

(1) Lightweight network design: Although ViT and ViM have demonstrated state-of-the-art performance in vision tasks, convolution-based architectures still hold great application prospects due to their lightweight nature. We believe that developing lightweight network structures, or hybrid models that combine the efficiency of CNNs with the high performance of ViT or ViM, hold great potential for achieving efficient and high-quality CGH reconstruction.
(2) Compact network design strategy: To tackle the computational demands and memory limitations of generating ultra-high-resolution (4K/8K) holograms, compact network strategy including low-bit quantization framework,^157^ pruning,^158^^,^^159^ knowledge distillation,^160^^,^^161^ and super-resolution^127^^,^^162^ algorithms can be effectively utilized to optimize the overall performance with limited parameters and flops.
(3) Joint training feasibility for multiple dimension input: Reconstructing holograms with multidimensional information (e.g., wavelength, propagation distance, and polarization states) may require a joint training framework capable of processing multiple parameters simultaneously within a unified network, rather than training separate models for individual input dimensions. Such an approach could improve computational efficiency while potentially enhancing reconstruction quality through inter-channel interaction mechanisms^163^^,^^164^ that exploit inherent correlations between physical parameters.

The integration of these approaches can effectively balance computational efficiency and model performance, significantly improving real-time processing capabilities while reducing computational cost.

### Perceptual fidelity with real world and practical applications

As a promising 3D display technology, the adage “Seeing is believing” holds particular relevance in DLCGH, where the ultimate goal is to achieve photorealistic representations of physical reality through deep learning. While simulation accuracy remains a fundamental concern, the critical challenge lies in developing neural network architectures that can bridge the theoretical predictions with empirical observations. This principle has garnered significant attention in recent research, with growing emphasis on perceptual fidelity and user-centric design.

From a human-computer interaction perspective, modern DLCGH models must fulfill two fundamental requirements derived from the inherent complexity of real-world scenes: true-to-life 3D reconstruction and full-color demonstration, although some works prioritize 2D or monochromatic outputs. Furthermore, while current approaches predominately employ layer-based approaches (e.g., RGBD/LDI datasets) for 3D reconstruction, the elements decomposition method of 3D scenes is not only layered slicing. Immersible stereoscopic visual cues can be provided through various forms of dataset and training method, which may become a popular issue in the future. Meanwhile, there has been a proliferation of hybrid frameworks integrating physical simulation with artificial intelligence to mitigate the theoretical-practical discrepancy in holography. These synergistic approaches aim to achieve both computational efficiency and visual authenticity, representing a pivotal trajectory toward realizing immersive holographic metaverses. We anticipate continued advancements in DLCGH, which will ultimately determine the feasibility of authentic holographic display interactive systems. These challenges are compounded by the complexity of real-world scenes, which require true-to-life 3D reconstruction and full-color demonstration—capabilities often constrained by current layer-based approaches (e.g., RGBD/LDI datasets) that oversimplify 3D scene decomposition. Emerging 3D reconstruction techniques like neural radiance fields (NeRF)^165^ and 3D Gaussian splatting^166^ offer real-time photorealistic rendering but lack integration with holography for full-parallax 3D displays.

Moreover, human-eye friendliness necessitates minimizing visual fatigue and adapting to dynamic viewing conditions. For instance, realizing high-fidelity performance using human eye-friendly LED/SLED light sources.^140^ Additionally, gaze-continuity optimization and pupil-adaptive mechanisms are critical for achieving 3D realism in near-eye displays. Recent works^167^^,^^168^^,^^169^^,^^170^^,^^171^ have demonstrated that incorporating parallax cues and adaptive pupil responses can enhance the visual experience and realism of holographic displays, especially in enhancing the display quality at defocused positions.

These efforts regarding perceptual fidelity with real world are a crucial step toward the practical application of CGH, as holographic display technology is tailored for human eyes with excellent visual abilities. Recent achievements have fully demonstrated the feasibility of leveraging deep learning techniques to significantly enhance the real-world reconstruction quality of holograms, proving it to be currently the most effective approach compared to other traditional algorithms. Furthermore, with the support of high-performance hardware computing devices and computational frameworks, DLCGH also represents the most promising approach for achieving real-time dynamic holographic display with high refresh rates. We anticipate that with continued synergistic innovation between deep learning and CGH, DLCGH will soon transition from the laboratory to practical applications in commercial consumer-grade displays in the foreseeable future.

## Acknowledgments

This work was funded by the 10.13039/501100001809National Natural Science Foundation of China (grant 62205117 and 52275429), 10.13039/501100012166National Key Research and Development Program of China (grant 2021YFF0502700), the Young Elite Scientists Sponsorship Program by CAST (grant 2022QNRC001), 10.13039/501100013494West Light Foundation of the Chinese Academy of Sciences (grant xbzg-zdsys-202206), Knowledge Innovation Program of Wuhan-Shuguang, Innovation project of Optics Valley Laboratory (grant OVL2021ZD002), 10.13039/501100003819Hubei Provincial Natural Science Foundation of China (grant 2022CFB792).

## Author contributions

Conceptualization, X.Y.; writing—original draft, X.Y. and H.Z.; writing—review and editing, X.Y., H.Z., Z.Z., X.F., S.H., Z.L., W.C., D.L., S.S., W.X., and H.G.; supervision, W.X. and H.G.; funding acquisition, W.X. and H.G. All authors have read and agreed to the published version of the manuscript.

## Declaration of interests

The authors declare no competing interests.
